# Benign appearing breast lesions age 30 and below: to biopsy, or not to biopsy?

**DOI:** 10.1186/bcr3301

**Published:** 2012-11-09

**Authors:** AC Nisbet, R Bradley

**Affiliations:** 1 NHS Lothian, Edinburgh, UK

## Introduction

Currently, most women under 30 with solid breast lumps undergo biopsy, despite classic benign features on ultrasound. However, breast cancer is uncommon in this age group and the vast majority have obvious sinister features on ultrasound. There is a growing argument that many of these younger women are being subjected to an unnecessary invasive test with obvious resource and financial implications.

## Methods

A retrospective review of images and reports of all the diagnosed breast cancers in women aged 30 and below in the Edinburgh area between 2006 and 2011.

## Results

A total of 37 cancers were detected in women of the sample age group. Of these, 35 had definable masses seen on ultrasound, all of which were considered R3 or above, and none of which on imaging review met benign criteria. The remaining two cancers were occult on ultrasound with no definable mass.

## Conclusion

Over a 5-year period, no breast cancers would have been missed in our unit if a policy not to biopsy classic benign solid breast lesions in women aged 30 and below had been implemented. Although only looking at small numbers, this study adds to the growing evidence that biopsies of lesions with classic benign features in women of this age group are extremely unlikely to result in malignant pathology, and that current policy to routinely biopsy may be unnecessary.

